# Antitumor Research of the Active Ingredients from Traditional Chinese Medical Plant Polygonum Cuspidatum

**DOI:** 10.1155/2018/2313021

**Published:** 2018-11-21

**Authors:** Xinnan Wu, Qi Li, Yu Feng, Qing Ji

**Affiliations:** Department of Medical Oncology and Cancer Institute of Integrative Medicine, Shuguang Hospital, Shanghai University of Traditional Chinese Medicine, Shanghai 201203, China

## Abstract

In recent years, the utilization of Chinese native medicine and other plant extracts in the treatment of diseases has attracted extensive attention, especially in the area of malignant tumors. However, lots of herbal remedies active ingredients have not been found or have been discovered but not effectively developed and applied. Therefore, screening new Chinese medicine active components and determining their antitumor effects have become a new breakthrough in the prevention and treatment of tumor disease. In the past years, a large number of studies have demonstrated that Polygonum cuspidatum and its active components like resveratrol showed excellent antitumor activities, including our own antitumor studies about resveratrol in colorectal cancer. The purpose of this review is to summarize the research progress of Chinese herb Polygonum cuspidatum and its active components in tumor diseases and provide theoretical basis for further scientific experiments and clinical applications.

## 1. Introduction

With the changes of the diet types and the bad living habits, malignant tumors have gradually developed into a serious threat to human health and life. In the United States, according to the current peak levels [1], from 1991 to 2014, overall cancer mortality dropped by 25%; however, in the past 2017 years, 1,688,780 new cancer cases and 600,920 cancer deaths are still expected to occur in USA. In 2015, 4292,000 new cancer cases and 2814,000 cancer deaths have been estimated to occur in China [2], according to the data released by the National Central Cancer Registry of China.

At present, the treatment of tumors mainly focuses on surgery, radiation therapy, chemotherapy, molecular targeted therapy, and immunotherapy. But these treatments, to some extent, easily produce side effects on normal cells, organs, and other tissues of the human body, thus accelerating the death process of cancer patients. Many natural products and their active components have been reported to have potential antitumor or tumor preventive properties. Therefore, the full utilization of some natural products and their active components will provide unique ideas and methods for cancer prevention and treatment. Polygonum cuspidatum Sieb. et Zucc., a Traditional Chinese medicine (TCM) herb, belongs to polygonaceae. It has a long history of being used as a folk medicine in China, Japan, and Korea. Pharmacological researches and clinical studies have indicated that Polygonum cuspidatum extraction and its major compounds possess antitumor [3], anti-inflammatory, antivirus, antimicrobial, neuroprotective, and cardioprotective activities [4]. Previous researches completed by our team have also demonstrated that resveratrol inhibits the proliferation, invasion, and metastasis of colorectal cancer cells [5]. Therefore, we believe that it is necessary to systematically summarize the antitumor effects of Polygonum cuspidatum and its active components and lay a foundation for their clinical development and application.

## 2. Antitumor Activities of Polygonum Cuspidatum Active Ingredients

**Figure 1:**
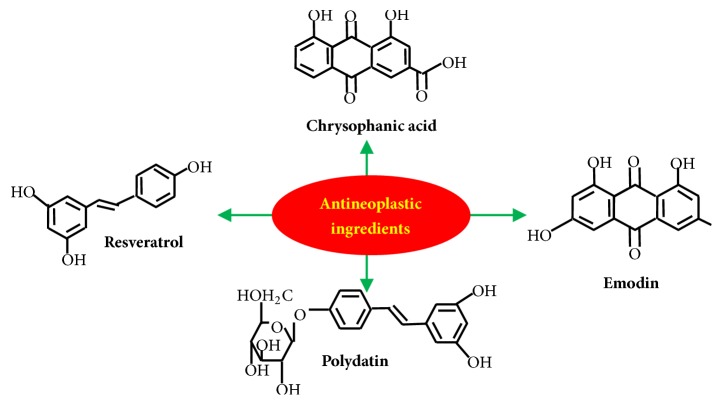
Antitumor components of Polygonum cuspidatum. Resveratrol, C14H12O3, trans-3t4, and 5-Trihydroxystilbene. Polydatin, C20H22O8, 3,4′-5-Trihydroxystilbene-3-beta-D-glucopyranoside. Emodin, C15H10O5, and 1,3,8-Trihydroxy-6-methylanthraquinone. Chrysophanic acid, C15H8O6, 4,5-dihydroxy-9,10-dioxo-9, and 10-dihydroanthracene-2-carboxylic acid.

Until now, many active constituents of Polygonum cuspidatum have been found, such as resveratrol, polydatin, and anthraquinones (including emodin and its glycoside). It also contains flavonoids such as quercetin and (+)-catechin [4]. A growing number of researches have shown that the effect of Polygonum cuspidatum and its active ingredients in cancer treatment is remarkable. Here we will review the research progress of active components of Polygonum cuspidatum in tumor diseases therapy, mainly including resveratrol, polydatin, emodin, and chrysophanic acid (Figure 1).

### 2.1. Antitumor Activity of Resveratrol

**Figure 2:**
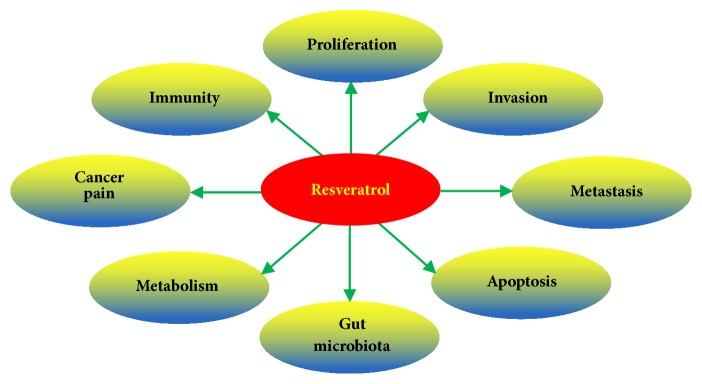
Antitumor effect of resveratrol. Resveratrol not only acts on tumor cells themselves but also regulates human immunity and microenvironment. Moreover, it can improve the life quality of cancer patients by improving cancer pain.

Resveratrol was originally extracted from the roots of Polygonum cuspidatum, which is also found in red wine, grapes, and peanuts [6]. Many health benefits have been linked to it, including antitumor [7], anti-inflammation [6], antioxidation [8], immunoregulation [9], and even gut microbiota-regulation [10]. Currently, resveratrol has attracted attention of researchers for its antitumor effect in a variety of human cancer cell lines through the regulation of various molecular targets [11]. Its antitumor roles cover almost all aspects of cancer, including tumor cell proliferation, invasion, metastasis, apoptosis [7], immunity [12], metabolism [13], and intestinal flora [14] (Figure 2).

#### 2.1.1. Resveratrol and Tumor Proliferation, Invasion, Metastasis, and Apoptosis

**Figure 3:**
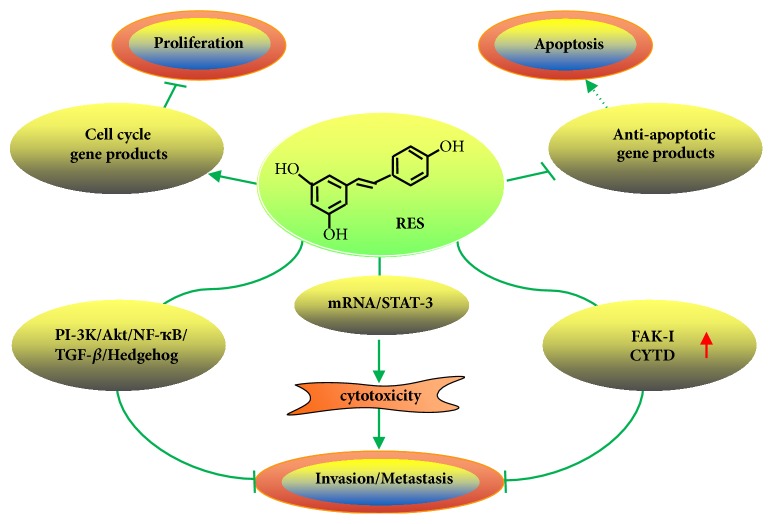
Effect of resveratrol on the proliferation, invasion, metastasis, and apoptosis of tumor cells. Resveratrol can restrain the proliferation of multiple cancer cells through modulation of cell-cycle regulatory gene products and induce the cancer cells apoptosis by inhibition of antiapoptotic gene products. Resveratrol can inhibit EMT associated cancer cell invasion and migration through the inhibition of the PI-3K/Akt/NF-ҡB, TGF-*β*1, and hedgehog signaling pathway. Resveratrol displayed a dose-dependent and time-dependent cytotoxicity on lung cancer cells A549 through inhibiting the mRNA and protein expression of STAT-3. Resveratrol can enhance the anti-invasion and antimetastasis effect of FAK-I and CYTD when they were used in combination.

In the past few years, resveratrol has been found to play important roles in tumor progression, including proliferation, invasion, metastasis, and apoptosis (Figure 3). Resveratrol is a potent natural activator of sirtuin-1 (SIRT1), a nuclear substance associated with the histone deacetylases class III [15]. Moreover, resveratrol can inhibit epithelial mesenchymal transition (EMT) associated cancer cell invasion and migration through the inhibition of the PI-3K/Akt/NF-ҡB, TGF-*β*1, and hedgehog signaling pathway [16–18]. As our previous experiments in vitro have shown, TGF-*β*1-induced EMT promoted the invasion and metastasis of colorectal cancer, but resveratrol could inhibit the invasive and migratory ability of LoVo cells in a concentration-dependent manner through regulating TGF-*β*1/Smads signaling pathway mediated Snail/E-cadherin expression [19].

Nuclear factor-kappa B (NF-ҡB) is a critical element, which regulates kinds of pathophysiological processes, including proliferation, invasion, metastasis, differentiation, and apoptosis of different tumor cells [20]. Resveratrol is a specific inhibitor of NF-ҡB in different tumor cells [21]; it can downregulate the nuclear localization of NF-ҡB phosphorylation and its acetylation, which cause attenuation of NF-ҡB-regulated gene products (MMP-9, CXCR4) involved in tumor-invasion and metastasis [22]. Resveratrol can also downregulate NF-ҡB signaling pathway by inhibiting activation of IҡB*α* kinase and IҡB*α* phosphorylation in colorectal cancer cells. Additionally, the regulation of intercellular junctions and EMT is one of the principle mechanisms of resveratrol on the inhibition of tumor growth and invasion [23]. Resveratrol can restrain the proliferation of multiple cancer cells through modulation of cell-cycle regulatory gene products and induce the cancer cells apoptosis by upregulation of p53 and inhibition of antiapoptotic gene products [24].

Resveratrol can inhibit the phosphorylation of focal adhesion kinase (FAK) in various cancer cell lines [25]. Moreover, resveratrol displayed a dose-dependent and time-dependent cytotoxicity on lung cancer cells A549 through inhibiting the mRNA and protein expression of STAT-3, while overexpression of STAT-3 completely or partially blocked the effects of resveratrol on A549 cells [26]. The recombination and reconfiguration of cytoskeleton are very important for the invasion and metastasis of cancer cells. FAK-I (FAK-inhibitor) and CYTD (cytochalasin D) can inhibit the invasion and metastasis effect of cancer cells, while resveratrol can enhance the anti-invasion and antimetastasis effect of FAK-I and CYTD when they were used in combination [27].

#### 2.1.2. Resveratrol and Immunity

It is well-understood that radiation therapy is one of the most important treatment methods for cancers [28]. However, radiation may damage the DNA, cells, and organs and cause side effects associated with antiproliferation, proinflammation, profibrosis, and even patients' immune system imbalance [29]. It has been reported that radiation-induced production and inflammation can be prevented with flavonoids, including phenols such as resveratrol.

Spleen is the largest immune organ in mammals. The maintenance of splenic lymphocytes plays an important role in the normal immune function. Studies have shown that resveratrol can protect the immune function of spleen, which is manifested in the fact that it can significantly reverse restraint-induced declines of spleen index and splenocyte number. In addition, resveratrol plays an important role in protecting spleen cell mitochondria from oxidative stress [30].

It is well-known that T lymphocytes play an important role in cellular immunity, such as killing target cells, reacting to specific antigens, and producing cytokines. In mature T lymphocytes, CD4^+^ and CD8^+^ T cells are two important subsets of immune regulation [30]. CD4^+^ and CD8^+^ T cells are essential for OX 40 agonist mediated tumor immune system [31]. The data has shown that resveratrol could increase the proportion and quantity of CD4^+^ T cells [30]. However, during the treatment of OX 40 agonist, supplementation of resveratrol could not maintain the antitumor immune function [28]. The effect of resveratrol on tumor growth and radiation-induced immune dysfunction is not significant, which may be affected by the bioavailability of resveratrol, since more than 50% of resveratrol is bioavailable in rodents and humans shortly after intake [28].

It is believed that the body's immunity system, including macrophages, neutrophils, and natural killer cells, decreases with age [32]. This adaptive change in immune function can lead to a state of immune deficiency and affect tumor immune responses. Therefore, restoring or maintaining antitumor immunity in elderly cancer patients can improve the efficacy of immunotherapy [30]. Calorie restriction is the most reliable way to maintain immune function in elderly patients. Nevertheless, because of the difficulty in maintaining calorie for a long time, it is necessary to consider the immune protection of tumors maintained by calorie mimics such as resveratrol during aging, and more experiments are needed to fully determine whether resveratrol can maintain immunity during aging to prevent tumorigenesis or inhibit cancer cells [28].

#### 2.1.3. Resveratrol and Gut Microbiota

Studies have shown that gut microflora has an impact on human health and disease, which involves the potential of drug targeting and metabolism. TCM may restore homeostasis in humans by regulating gut microbes and restore metabolic/immune homeostasis by modulating genes within the host [33]. This will be of great help to the prevention and treatment of various types of bowel cancers [14].

Several mechanisms of resveratrol have been proposed, including modulating the gut microbiotacan, gut integrity, and barrier function [10]. The effect of resveratrol on the intestinal flora is primarily that it preferentially slows down the growth of certain microbes, leading to a more favorable microbial distribution [10]. In addition, resveratrol metabolites produced by gut microflora have distinct biological effects, which may have guiding significance for the study of digestive tract tumors [10]. The physiological effects of resveratrol are in striking contrast to its low bioavailability, which is a major problem for the development of the kind of compounds into therapeutic agents. However, the evidence supports the opinion that phenolic phytochemicals with low bioavailability are possibly playing a role through remodeling the gut microbiota [34]. All above results suggested that resveratrol can significantly modulate the gut microbiota to improve intestinal microenvironment and further prevent the occurrence and development of tumors [35].

#### 2.1.4. Resveratrol and Metabolism

**Figure 4:**
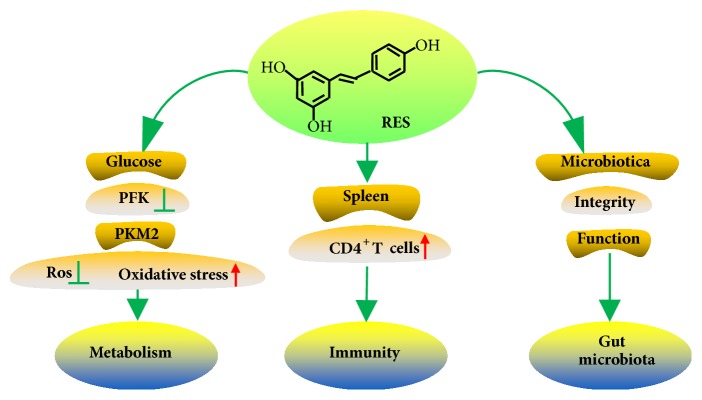
Effect of resveratrol on immunity, gut microbiota, and metabolism. Resveratrol could protect spleen immune function and reverse the decrease of spleen index and spleen cell number. Resveratrol could increase the proportion and quantity of CD4^+^ T cells. It improves the intestinal environment, including modulating the gut microbiotacan, gut integrity, and barrier function. The anticancer mechanism of resveratrol is related to the regulation of glucose metabolism. It inhibits the metabolism of tumor by affecting the state of PKM2 and decreases intracellular reactive oxygen species production and oxidative stress through mechanisms involving degradation of Keap 1 protein, which is a repressor of Nrf2.

Like normal cells, metabolism is necessary for cancer cells to generate energy for promoting cell proliferation [36]. Increased glucose uptake and lactate production are marks of cancer metabolism [37]. The effect mechanism of resveratrol on cancer metabolism has been found in several aspects (Figure 4). Firstly, the regulation of glucose transporter (GLUT) and glycolytic enzyme activity by AKT is one of the mechanisms of metabolic phenotype in cancer cells [38], and resveratrol can regulate the glucose metabolism by blocking the transport of GLUT1 to the plasma membrane via inhibiting the activation of AKT [39]. Secondly, the inhibition of enzyme 6-phosphofructo-1-kinase (PFK) could result in the death of human breast cancer cell lines and tissues [40], while resveratrol could directly inhibit the activity of purified PFK, thus providing a new target for the antibreast tumor [41]. Thirdly, the pyruvate kinase M2 (PKM2) is the key to tumor metabolism and growth [42], and the resveratrol can inhibit cancer metabolism by affecting the state of PKM2 [43]. Furthermore, mitochondrial dysfunction associated with tumors leads to a significant increase in reactive oxygen species (Ros) production [44], but resveratrol can inhibit reactive oxygen species and reduce oxidative stress through the degradation of Keap 1 protein, which is a repressor of Nrf2 [45].

#### 2.1.5. Resveratrol and Inflammation

Inflammation has been considered to be a “hallmark of cancer” [46]. Epidemiological and clinical studies have made it clear that about 25% of solid tumors are associated with chronic inflammation [47]. In cancer, inflammation is a continuous process, and persistent chronic inflammatory response will promote tumor proliferation, angiogenesis, invasion, and metastasis. In addition, inflammation, EMT, endoplasmic reticulum (ER) stress, and metabolism often interact with each other, affecting the occurrence and development of tumor [48].

**Figure 5:**
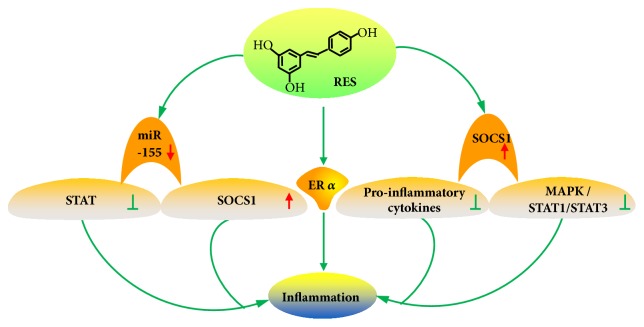
Resveratrol and inflammation. Resveratrol inhibits STAT activation and enhances SOCS1 expression by attenuating the production of miR-155. Resveratrol inhibits the production of proinflammatory cytokines and inhibits the activation of the p38 MAPK and STAT1/STAT3 signaling pathways by upregulating SOCS1 expression. The anti-inflammatory response of resveratrol was related to the binding of ER*α*.

Several reports have shown the important regulatory effect of resveratrol on inflammation through different targets and various signaling pathways (Figure 5). Suppressor of cytokine signaling 1 (SOCS1) is typically perceived as a tumor suppressor, and silencing of the SOCS1 gene by hypermethylation in its promoter region is frequent in many types of cancer. However, the role of SOCS1 in colorectal cancer has been poorly investigated [49]. Resveratrol exerts anti-inflammatory effects through the upregulation of SOCS1, which is a potential target of miR-155. At the same time, resveratrol inhibits STAT activation and enhances SOCS1 expression by attenuating the production of miR-155. These findings suggest that resveratrol may be developed as a useful agent for the treatment of inflammatory diseases. In addition, resveratrol inhibits the production of proinflammatory cytokines and inhibits the activation of the p38 mitogen-activated protein kinase (MAPK) and STAT1/STAT3 signaling pathways by upregulating SOCS1 expression in response to LPS stimulation [50].

Estrogen receptor-*α* (ER*α*) is an important transcription factor that modulates cell growth in various tissues [51], which is closely associated with the development of multiple cancers, especially endometrial carcinoma and breast cancer. Nwachukwu et al. have found that the anti-inflammatory response of resveratrol was related to the binding of ER*α*, which changes the shape of the receptor through the coregulator molecules to regulate transcription [52]. Resveratrol is a transduction selective ER*α* ligand, which adjusts the inflammatory response without stimulating proliferation by dynamically binding with the receptor and induces an altered activation function 2 coactivator-binding site. In addition, it also regulates the recruitment of a cast of coregulators at the IL-6 locus [6].

#### 2.1.6. Resveratrol and Cancer Pain

Cancer pain is one of the most common clinical symptoms associated with malignant cancers [53]. Nowadays, opioids are used to treat moderate to severe pain. Among them, morphine is an effective analgesic for treating moderate to severe pain [54]. However, long-term morphine administration induces tolerance [54] and robust activation of spinal microglia and even resulted in a marked reduction in the analgesic properties [55], which hampers its clinical use. Therefore, it is urgent to treat cancer pain safely and effectively.

**Figure 6:**
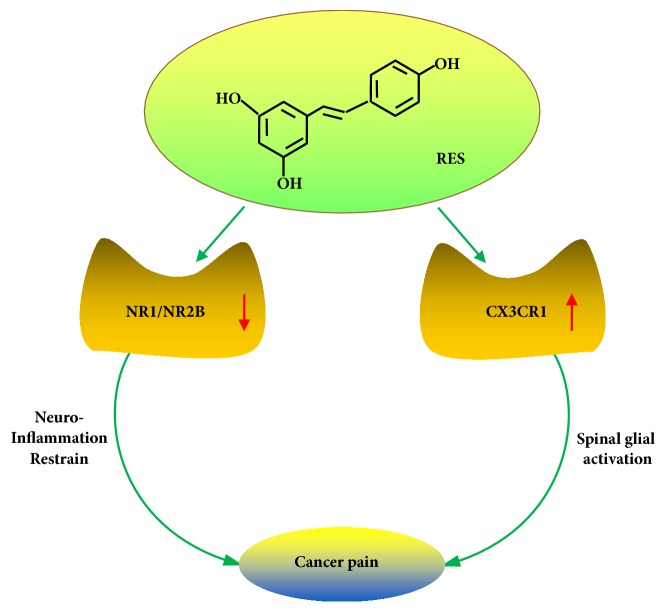
Resveratrol and cancer pain. Resveratrol can mitigate morphine tolerance through restraining neuroinflammation and downregulating NMDAR NR1 and NR2B subunit expression. The suppression of spinal glial activation and CX3CR1 upregulation is another mechanism of the analgesic effects of resveratrol.

Resveratrol possesses potentially analgesic effects [56], and it has no known toxic side effects. Therefore, resveratrol may constitute an effective, safe, and convenient treatment option for cancer patients experiencing severe pain (Figure 6). Long-term morphine infusion induced N-methyl-D-aspartate receptor (NMDAR) NR1 and NR2B subunit upregulation in synaptosomal membrane of morphine-tolerant rat lumbar spinal cords, which was suppressed by the resveratrol treatment [54]. Resveratrol can mitigate morphine tolerance through restraining neuroinflammation and downregulating NR1 and NR2B expression [54]. Someone also found out reduction of postsynaptic membrane PSD-95 (postsynaptic density-95) NMDAR expression by resveratrol treatment may be responsible for attenuating glial activation in morphine-tolerant rat spinal cords [54].

Researchers have unearthed that resveratrol can significantly inhibit the morphine-induced microglia cell activation and migration. The suppression of spinal glial activation and CX3C chemokine receptor 1 (CX3CR1) upregulation is another mechanism of the analgesic effects of resveratrol [55]. At present, resveratrol could delay and attenuate cancer-induced pain facilitation through intrathecal administration, and resveratrol could also attenuate cancer pain induced CX3CR1 upregulation and glial activation in the spine [55].

### 2.2. Antitumor Activity of Polydatin

**Figure 7:**
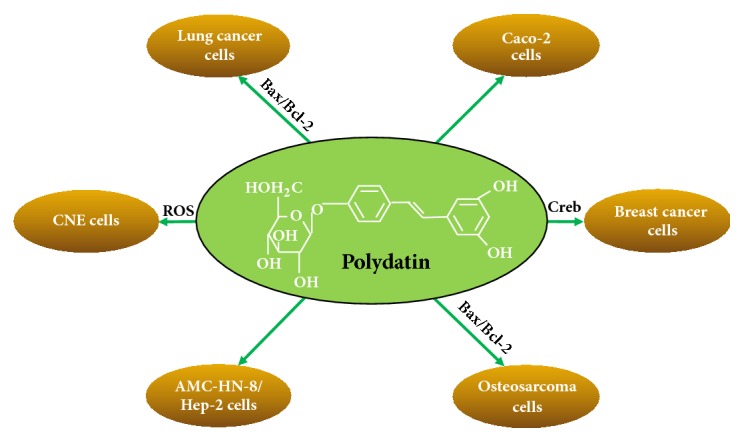
Cell selectivity of polydatin. Polydatin induces apoptosis effectively with an increase in Bax expression and a decrease in Bcl-2 expression in lung cancer cells. Polydatin has a significant time- and dose-dependent inhibitory effect on the proliferation inhibition and apoptosis induction of HCC cells. Polydatin can induce apoptosis of human osteosarcoma cells by upregulation of the ratio of Bax/Bcl-2. Polydatin can induce the production of ROS which triggers ER stress and mitochondrial apoptotic pathways in CNE cells. It has a strong cytotoxicity to the growing Caco-2 cells. Polydatin can inhibit the activation of Creb, and the aim of inhibiting the proliferation of breast cancer cells.

Polydatin is a stilbenoid compound isolated from the root of Polygonum cuspidatum [57], as resveratrol derivative with a glucopyranoside ring substitution of the hydroxyl group in position three, has higher stability, has water solubility, and is more resistant to enzymatic oxidation, and even has a strong cytotoxicity, and is able to enter cells via glucose transporters [58–60]. It is precisely because of these characteristics that polydatin has greater bioavailability than resveratrol and thus has a better preventive and therapeutic effect on cancer (Figure 7).

As a rule, apoptosis is regulated by proapoptotic and antiapoptotic proteins of the Bcl-2 family and is executed through caspases or cysteine-aspartic proteases [57]. The results of the present study showed that polydatin induces apoptosis effectively with an increase in Bax expression and a decrease in Bcl-2 expression in lung cancer cells, providing a theoretical basis for the prevention and treatment of lung cancer by polydatin. In other studies, polydatin has a significant time- and dose-dependent inhibitory effect on the proliferation inhibition and apoptosis induction of HCC cells [61]. In addition, polydatin can induce apoptosis of human osteosarcoma cells by upregulation of the ratio of Bax/Bcl-2. Reactive oxygen species are mediators of intracellular signaling cascades that can induce apoptosis associated with mitochondria. Excessive production of Ros triggers oxidative stress, loss of cell function, and even apoptosis [62]. At the same time, polydatin can promote the apoptosis through inducing the production of Ros which triggers endoplasmic reticulum (ER) stress and mitochondrial apoptotic pathways in human nasopharyngeal carcinoma CNE cells [60].

In mammalian, the core cell-cycle mechanism comprising cyclin and cyclin-dependent kinase complex is the main cause of cell proliferation [63]. D-type cyclins are typical targets and crucial signaling molecules for cancer treatment [64]. Among them, cyclin D1 is an importantly regulatory factor in cell-cycle progression, and it plays a transcriptional coregulator role [65]. Cyclin D1 is necessary for tumor maintenance [66], and cell-cycle regulation is an effective strategy for inhibiting tumor growth [67]. Polydatin shows its antiproliferation effect by inhibiting the expression of cyclin D1 and cyclin B1, resulting in cell-cycle arrest in S-phase [66].

CAMP response element-binding proteins (Creb) are a characteristic transcription element of the leucine zipper family [68]. Creb is a significant factor affecting different solid tumors genesis and metastasis. For example, in breast cancer patients with a poor prognosis, metastatic disease, and nodal involvement [69], the level of Creb 1 was significantly upregulated. A report has demonstrated that polydatin can significantly reduce the phosphorylation level of Creb in a dose-dependent manner, leading to the inactivation of Creb, followed by the proliferation inhibition of breast cancer cells [70].

In previous studies, polydatin, lonely or in combination with resveratrol, was found to inhibit growth and differentiation of Caco-2 cells [59]. Compared with resveratrol, polydatin has better cell selectivity. It has a strong cytotoxicity to the growing Caco-2 cells, and its toxicity is about 3 times lower in the differentiated Caco-2 cell [59]. In addition, the selectivity of polydatin is also reflected in human nasopharyngeal carcinoma CNE cells. For instance, it can induce the production of reactive oxygen species to trigger ER stress and mitochondrial apoptotic pathways [60]. All these results suggest that polydatin plays a cytotoxic role through the mechanisms that are different from resveratrol.

### 2.3. Antitumor Studies of other Active Components from Polygonum Cuspidatum

**Figure 8:**
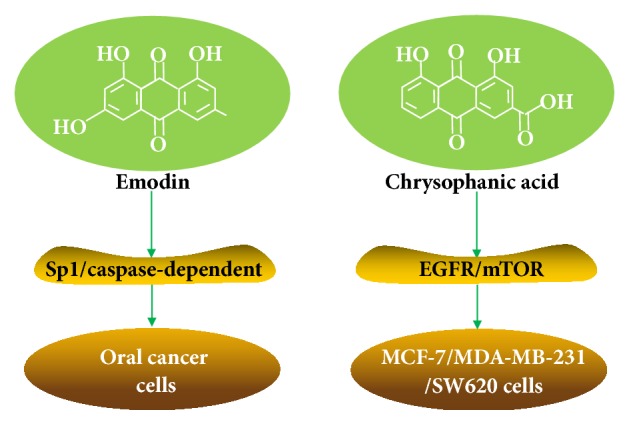
Antitumor effects of emodin and chrysophanic acid. Emodin can inhibit the growth of oral cancer cells by reducing Sp1 and inducing caspase-dependent apoptosis. Chrysophanic acid has anticancer activity through its effect on EGFR/mTOR mediated signaling transduction pathway.

Some other active components from Polygonum cuspidatum have been found to have antitumor activities, such as emodin and chrysophanic acid (Figure 8). Emodin, as one of the main active components of Polygonum cuspidatum, has anti-inflammatory and antioxidative [71], antimicrobial [72], and antitumor effects [73] as resveratrol. Emodin has been shown to have strong antitumor activity in oral cancer cells. It can inhibit the growth of oral cancer cells by reducing specificity protein 1 (Sp1) and induce caspase-dependent apoptosis, suggesting that emodin may be a potential bioactive substance to induce apoptosis [74].

Chrysophanic acid, another active component of Polygonum cuspidatum, has antiproliferative effect on human breast cancer cells MCF-7 and MDA-MB-231, as well as the human colon cancer cells SW620 [75, 76]. Chrysophanic acid also inhibits the activation of EGF-induced epidermal growth factor receptor (EGFR) and the downstream signaling molecules, including AKT, mTOR, and ribosomal protein S6 kinase. These findings indicate that chrysophanic acid has anticancer activity through its effect on EGFR/mTOR mediated signaling transduction pathway. In addition, combined application of chrysophanol acid and mTOR inhibitors may enhance the antiproliferation effect [77].

## 3. Problems and Prospects

In last several years, the good curative effect of TCM in treating some difficult diseases has been widely acknowledged. Polygonum cuspidatum, as a kind of herbs, is rich in resources and widely used in clinical practice of TCM treatment. Modern studies have shown that Polygonum cuspidatum has a remarkable curative effect in the prevention and treatment of tumor diseases.

However, there are still many problems in the development and utilization of Polygonum cuspidatum or even other Chinese herbal medicines. Firstly, the current technology is insufficient to completely explain the complex composition of herbal medicines. There are hundreds of active ingredients in natural plant medicines; however, due to the limitations of current science and technology, only a part of the active ingredients can be extracted and characterized. The limited efficacy and scope of application of these extracts may be related to the undiscovered active ingredients in the same plant. Secondly, as a worldwide disease, the pathogenesis of tumors is not well-understood. Therefore, in the targeted therapy, the effects of the active ingredients from TCM are not fully exerted. For example, if the concentration of some enzymes in the tumor microenvironment can be determined, the concentration of enzymes would be selectively increased to enhance the bioavailability of herbs and their active components. Thirdly, at present, the screening techniques are faultiness on the effective components of the TCM. At the same time, it is well-known that the medicinal effect of the TCM has a great relationship with the dosage. We should constantly develop new technology, strengthen the identification of new active components, and clarify the mechanism of TCM and its active components in order to improve the bioavailability of drugs. In addition, the signal pathway relationship between components of TCM is not supported by enough data. Different modifications may lead to different target pathways and changes in cellular activity. Therefore, it is necessary to further study the relationship between herbs components and signal transduction pathway. Last but not least, a lot of malignant cancer patients have short survival time and earlier intervention in tumor treatment, which caused great disturbance and uncertainty for the clinical research of TCM and its effective components.

In future research, we should intensify the research on the clinical trials, to eliminate the interference factors on curative effect observation and improve the overall therapeutic effectiveness of cancer patients.

## References

[1] Siegel R. L., Miller K. D., Jemal A. (2017). Cancer statistics, 2017. *CA: A Cancer Journal for Clinicians*.

[2] Chen W., Zheng R., Baade P. D. (2016). Cancer statistics in China, 2015. *CA: A Cancer Journal for Clinicians*.

[3] Zhang L., Li Y., Gu Z. (2015). Resveratrol inhibits enterovirus 71 replication and pro-inflammatory cytokine secretion in rhabdosarcoma cells through blocking IKKs/NF-*κ*B signaling pathway. *PLoS ONE*.

[4] Zhang H., Li C., Kwok S.-T., Zhang Q.-W., Chan S.-W. (2013). A review of the pharmacological effects of the dried root of *Polygonum cuspidatum* (Hu Zhang) and its constituents. *Evidence-Based Complementary and Alternative Medicine*.

[5] Ji Q., Liu X., Fu X. (2013). Resveratrol inhibits invasion and metastasis of colorectal cancer cells via MALAT1 mediated Wnt/*β*-catenin signal pathway. *PLoS ONE*.

[6] Nwachukwu J. C., Srinivasan S., Bruno N. E., Parent A. A., Hughes T. S., Pollock J. A. (2014). Resveratrol modulates the inflammatory response via an estrogen receptor-signal integration network. *Elife*.

[7] Han G., Xia J., Gao J., Inagaki Y., Tang W., Kokudo N. (2015). Anti-tumor effects and cellular mechanisms of resveratrol. *Drug discoveries & therapeutics*.

[8] Jeong S. I., Shin J. A., Cho S. (2016). Resveratrol attenuates peripheral and brain inflammation and reduces ischemic brain injury in aged female mice. *Neurobiology of Aging*.

[9] Kowalska A., Siwicki A. K., Kowalski R. K. (2017). Dietary resveratrol improves immunity but reduces reproduction of broodstock medaka Oryzias latipes (Temminck & Schlegel). *Fish Physiology and Biochemistry*.

[10] Bird J. K., Raederstorff D., Weber P., Steinert R. E. (2017). Cardiovascular and antiobesity effects of resveratrol mediated through the gut microbiota. *Advances in Nutrition*.

[11] Ko J., Sethi G., Um J. (2017). The role of resveratrol in cancer therapy. *International Journal of Molecular Sciences*.

[12] Chen L., Yang S., Liao W., Xiong Y. (2015). Modification of antitumor immunity and tumor microenvironment by resveratrol in mouse renal tumor model. *Cell Biochemistry and Biophysics*.

[13] Pieszka M., Szczurek P., Ropka-Molik K., Oczkowicz M., Pieszka M. (2016). The role of resveratrol in the regulation of cell metabolism - A review. *Postepy Higieny i Medycyny Doswiadczalnej*.

[14] Chen F., Wen Q., Jiang J. (2016). Could the gut microbiota reconcile the oral bioavailability conundrum of traditional herbs?. *Journal of Ethnopharmacology*.

[15] Moore R. L., Dai Y., Faller D. V. (2012). Sirtuin 1 (SIRT1) and steroid hormone receptor activity in cancer. *Journal of Endocrinology*.

[16] Li W., Ma J., Ma Q. (2013). Resveratrol inhibits the epithelial-mesenchymal transition of pancreatic cancer cells via suppression of the PI-3K/Akt/NF-*κ*B pathway. *Current Medicinal Chemistry*.

[17] Wang H., Zhang H., Tang L. (2013). Resveratrol inhibits TGF-*β*1-induced epithelial-to-mesenchymal transition and suppresses lung cancer invasion and metastasis. *Toxicology*.

[18] Li J., Chong T., Wang Z. (2014). A novel anti-cancer effect of resveratrol: Reversal of epithelial- mesenchymal transition in prostate cancer cells. *Molecular Medicine Reports*.

[19] Ji Q., Liu X., Han Z. F. (2015). Resveratrol suppresses epithelial-to-mesenchymal transition in colorectal cancer through TGF-*β*1/Smads signaling pathway mediated Snail/E-cadherin expression. *BMC Cancer*.

[20] Chung J. S., Lee S., Yoo Y. D. (2014). Constitutive NF-*κ*B activation and tumor-growth promotion by Romo1-mediated reactive oxygen species production. *Biochemical and Biophysical Research Communications*.

[21] Benitez D. A., Hermoso M. A., Pozo-Guisado E., Fernández-Salguero P. M., Castellón E. A. (2009). Regulation of cell survival by resveratrol involves inhibition of NF*κ*B-regulated gene expression in prostate cancer cells. *The Prostate*.

[22] Buhrmann C., Shayan P., Popper B., Goel A., Shakibaei M. (2016). Sirt1 is required for resveratrol-mediated chemopreventive effects in colorectal cancer cells. *Nutrients*.

[23] Buhrmann C., Shayan P., Kraehe P., Popper B., Goel A., Shakibaei M. (2015). Resveratrol induces chemosensitization to 5-fluorouracil through up-regulation of intercellular junctions, epithelial-to-mesenchymal transition and apoptosis in colorectal cancer. *Biochemical Pharmacology*.

[24] Androutsopoulos V. P., Ruparelia K. C., Papakyriakou A., Filippakis H., Tsatsakis A. M., Spandidos D. A. (2011). Anticancer effects of the metabolic products of the resveratrol analogue, DMU-212: Structural requirements for potency. *European Journal of Medicinal Chemistry*.

[25] Vanamala J., Radhakrishnan S., Reddivari L., Bhat V. B., Ptitsyn A. (2011). Resveratrol suppresses human colon cancer cell proliferation and induces apoptosis via targeting the pentose phosphate and the talin-FAK signaling pathways—a proteomic approach. *Proteome Science*.

[26] Li X., Wang D., Zhao Q. C., Shi T., Chen J. (2016). Resveratrol inhibited non–small cell lung cancer through inhibiting STAT-3 signaling. *The American Journal of the Medical Sciences*.

[27] Stokes J. B., Adair S. J., Slack-Davis J. K. (2011). Inhibition of focal adhesion kinase by PF-562,271 inhibits the growth and metastasis of pancreatic cancer concomitant with altering the tumor microenvironment. *Molecular Cancer Therapeutics*.

[28] Farazi M., Nguyen J., Goldufsky J. (2014). Caloric restriction maintains OX40 agonist-mediated tumor immunity and CD4 T cell priming during aging. *Cancer Immunology, Immunotherapy*.

[29] Kim K.-O., Park H., Chun M., Kim H.-S. (2014). Immunomodulatory effects of high-protein diet with resveratrol supplementation on radiation-induced acute-phase inflammation in rats. *Journal of Medicinal Food*.

[30] Duan W.-J., Liu F.-L., He R.-R. (2013). Autophagy is involved in the effects of resveratrol on prevention of splenocyte apoptosis caused by oxidative stress in restrained mice. *Molecular Nutrition & Food Research*.

[31] Kjaergaard J., Tanaka J., Kim J. A., Rothchild K., Weinberg A., Shu S. (2000). Therapeutic efficacy of OX-40 receptor antibody depends on tumor immunogenicity and anatomic site of tumor growth. *Cancer Research*.

[32] Kovacs E. J., Palmer J. L., Fortin C. F., Fülöp T., Goldstein D. R., Linton P.-J. (2009). Aging and innate immunity in the mouse: impact of intrinsic and extrinsic factors. *Trends in Immunology*.

[33] Zhao L., Nicholson J. K., Lu A. (2012). Targeting the human genome-microbiome axis for drug discovery: Inspirations from global systems biology and traditional Chinese medicine. *Journal of Proteome Research*.

[34] Shin N.-R., Lee J.-C., Lee H.-Y. (2014). An increase in the *Akkermansia* spp. population induced by metformin treatment improves glucose homeostasis in diet-induced obese mice. *Gut*.

[35] Chen M.-L., Yi L., Zhang Y. (2016). Resveratrol attenuates trimethylamine-N-oxide (TMAO)-induced atherosclerosis by regulating TMAO synthesis and bile acid metabolism via remodeling of the gut microbiota. *mBio*.

[36] Ward P. S., Thompson C. B. (2012). Metabolic reprogramming: a cancer hallmark even warburg did not anticipate. *Cancer Cell*.

[37] Kato Y., Maeda T., Suzuki A., Baba Y. (2018). Cancer metabolism: New insights into classic characteristics. *Japanese Dental Science Review*.

[38] Cairns R. A., Harris I. S., Mak T. W. (2011). Regulation of cancer cell metabolism. *Nature Reviews Cancer*.

[39] Gwak H., Haegeman G., Tsang B. K., Song Y. S. (2015). Cancer-specific interruption of glucose metabolism by resveratrol is mediated through inhibition of Akt/GLUT1 axis in ovarian cancer cells. *Molecular Carcinogenesis*.

[40] Coelho R. G., Calaça I. D. C., Celestrini D. D. M., Correia A. H., Costa M. A. S. M., Sola-Penna M. (2011). Clotrimazole disrupts glycolysis in human breast cancer without affecting non-tumoral tissues. *Molecular Genetics and Metabolism*.

[41] Gomez L. S., Zancan P., Marcondes M. C. (2013). Resveratrol decreases breast cancer cell viability and glucose metabolism by inhibiting 6-phosphofructo-1-kinase. *Biochimie*.

[42] Beinat C., Alam I. S., James M. L., Srinivasan A., Gambhir S. S. (2017). Development of [(18)F]DASA-23 for imaging tumor glycolysis through noninvasive measurement of pyruvate kinase M2. *Molecular Imaging and Biology*.

[43] Iqbal M. A., Bamezai R. N. K. (2012). Resveratrol inhibits cancer cell metabolism by down regulating pyruvate kinase M2 via inhibition of Mammalian target of Rapamycin. *PLoS ONE*.

[44] Benfeitas R., Uhlen M., Nielsen J., Mardinoglu A. (2017). New challenges to study heterogeneity in cancer redox metabolism. *Frontiers in Cell and Developmental Biology*.

[45] Liu Y., Chan F., Sun H. (2011). Resveratrol protects human keratinocytes HaCaT cells from UVA-induced oxidative stress damage by downregulating Keap1 expression. *European Journal of Pharmacology*.

[46] Hanahan D., Weinberg R. A. (2011). Hallmarks of cancer: the next generation. *Cell*.

[47] Coussens L. M., Werb Z. (2002). Inflammation and cancer. *Nature*.

[48] Liu J., Lin P. C., Zhou B. P. (2015). Inflammation fuels tumor progress and metastasis. *Current Pharmaceutical Design*.

[49] Beaurivage C., Champagne A., Tobelaim W. S., Pomerleau V., Menendez A., Saucier C. (2016). SOCS1 in cancer: An oncogene and a tumor suppressor. *Cytokine*.

[50] Pfluger P. T., Herranz D., Velasco-Miguel S., Serrano M., Tschöp M. H. (2008). Sirt1 protects against high-fat diet-induced metabolic damage. *Proceedings of the National Acadamy of Sciences of the United States of America*.

[51] Zhou W., Slingerland J. M. (2014). Links between oestrogen receptor activation and proteolysis: relevance to hormone-regulated cancer therapy. *Nature Reviews Cancer*.

[52] Nwachukwu J. C., Southern M. R., Kiefer J. R. (2013). Improved crystallographic structures using extensive combinatorial refinement. *Structure*.

[53] Mantyh P. (2013). Bone cancer pain: causes, consequences, and therapeutic opportunities. *PAIN*.

[54] Tsai R.-Y., Chou K.-Y., Shen C.-H. (2012). Resveratrol regulates N-methyl-D-aspartate receptor expression and suppresses neuroinflammation in morphine-tolerant rats. *Anesthesia & Analgesia*.

[55] Cheng W., Zhao Y., Liu H. (2014). Resveratrol attenuates bone cancer pain through the inhibition of spinal glial activation and CX3CR1 upregulation. *Fundamental & Clinical Pharmacology*.

[56] Wang G., Hu Z., Song X. (2017). Analgesic and anti-inflammatory activities of resveratrol through classic models in mice and rats. *Evidence-Based Complementary and Alternative Medicine*.

[57] Zhang Y., Zhuang Z., Meng Q., Jiao Y., Xu J., Fan S. (2014). Polydatin inhibits growth of lung cancer cells by inducing apoptosis and causing cell cycle arrest. *Oncology Letters*.

[58] Krasnow M. N., Murphy T. M. (2004). Polyphenol glucosylating activity in cell suspensions of grape (Vitis vinifera). *Journal of Agricultural and Food Chemistry*.

[59] De Maria S., Scognamiglio I., Lombardi A. (2013). Polydatin, a natural precursor of resveratrol, induces cell cycle arrest and differentiation of human colorectal Caco-2 cell. *Journal of Translational Medicine*.

[60] Liu H., Zhao S., Zhang Y. (2011). Reactive oxygen species-mediated endoplasmic reticulum stress and mitochondrial dysfunction contribute to polydatin-induced apoptosis in human nasopharyngeal carcinoma CNE cells. *Journal of Cellular Biochemistry*.

[61] Jiao Y., Wu Y., Du D. (2018). Polydatin inhibits cell proliferation, invasion and migration, and induces cell apoptosis in hepatocellular carcinoma. *Brazilian Journal of Medical and Biological Research*.

[62] Zhang Y., Xiong Y., Zhou J., Xin N., Zhu Z., Wu Y. (2018). FoxO1 expression in osteoblasts modulates bone formation through resistance to oxidative stress in mice. *Biochemical and Biophysical Research Communications*.

[63] Malumbres M., Barbacid M. (2009). Cell cycle, CDKs and cancer: a changing paradigm. *Nature Reviews Cancer*.

[64] Wang H., Nicolay B. N., Chick J. M. (2017). The metabolic function of cyclin D3-CDK6 kinase in cancer cell survival. *Nature*.

[65] Kamarajugadda S., Becker J. R., Hanse E. A. (2016). Cyclin D1 represses peroxisome proliferator-activated receptor alpha and inhibits fatty acid oxidation. *Oncotarget *.

[66] Cao W.-J., Wu K., Wang C., Wan D.-M. (2016). Polydatin-induced cell apoptosis and cell cycle arrest are potentiated by Janus kinase 2 inhibition in leukemia cells. *Molecular Medicine Reports*.

[67] Funamizu N., Lacy C. R., Fujita K. (2012). Tetrahydrouridine inhibits cell proliferation through cell cycle regulation regardless of cytidine deaminase expression levels. *PLoS ONE*.

[68] Liu Y., Guo Y.-L., Zhou S.-J. (2010). CREB is a positive transcriptional regulator of gamma interferon in latent but not active tuberculosis infections. *Clinical and Vaccine Immunology*.

[69] Chhabra A., Fernando H., Watkins G., Mansel R. E., Jiang W. G. (2007). Expression of transcription factor CREB1 in human breast cancer and its correlation with prognosis. *Oncology Reports*.

[70] Chen S., Tao J., Zhong F. (2017). Polydatin down-regulates the phosphorylation level of Creb and induces apoptosis in human breast cancer cell. *PLoS ONE*.

[71] Tian S., Yang Y., Liu X., Xu Q. (2018). Emodin attenuates bleomycin-induced pulmonary fibrosis via anti-inflammatory and anti-oxidative activities in rats. *Medical Science Monitor*.

[72] Li L., Song X., Yin Z. (2016). The antibacterial activity and action mechanism of emodin from Polygonum cuspidatum against Haemophilus parasuis in vitro. *Microbiological Research*.

[73] Lin W.-F., Wang C., Ling C.-Q. (2015). Research progress in anti-tumor effect of emodin. *China journal of Chinese materia medica*.

[74] Shin J.-A., Shim J.-H., Jeon J.-G. (2011). Apoptotic effect of Polygonum Cuspidatum in oral cancer cells through the regulation of specificity protein 1. *Oral Diseases*.

[75] Choi S.-G., Kim J., Sung N.-D. (2007). Anthraquinones, Cdc25B phosphatase inhibitors, isolated from the roots of Polygonum multiflorum Thunb. *Natural Product Research (Formerly Natural Product Letters)*.

[76] Kang S. C., Lee C. M., Choung E. S. (2008). Anti-proliferative effects of estrogen receptor-modulating compounds isolated from *Rheum palmatum*. *Archives of Pharmacal Research*.

[77] Lee M. S., Cha E. Y., Sul J. Y., Song I. S., Kim J. Y. (2011). Chrysophanic acid blocks proliferation of colon cancer cells by inhibiting EGFR/mTOR pathway. *Phytotherapy Research*.

